# *Aedes aegypti* from temperate regions of South America are highly competent to transmit dengue virus

**DOI:** 10.1186/1471-2334-13-610

**Published:** 2013-12-28

**Authors:** Ricardo Lourenço-de-Oliveira, Anubis Vega Rua, Darío Vezzani, Gabriela Willat, Marie Vazeille, Laurence Mousson, Anna Bella Failloux

**Affiliations:** 1 Laboratório de Transmissores de Hematozoários, Instituto Oswaldo Cruz, Fiocruz, Av. Brasil 4365, Rio de Janeiro 21045-900, Brazil; 2 Department of Virology, Arboviruses and Insect Vectors, Institut Pasteur, Paris, France; 3 CONICET Instituto de Ecología, Genética y Evolución de Buenos Aires, Universidad de Buenos Aires, Buenos Aires, Argentina; 4 Unidad de Zoonosis y Vectores, Dirección General de la Salud, Ministerio de Salud Pública, Montevideo, Uruguay

**Keywords:** Vector competence, Experimental infections, Argentina, Uruguay, Dengue

## Abstract

**Background:**

*Aedes aegypti* is extensively spread throughout South America where it has been responsible for large dengue epidemics during the last decades. Intriguingly, dengue transmission has not been reported in Uruguay and is essentially prevalent in subtropical northern Argentina which borders Uruguay.

**Methods:**

We assessed vector competence for dengue virus (DENV) of *Ae. aegypti* populations collected in subtropical Argentina (Corrientes) as well as temperate Uruguay (Salto) and Argentina (Buenos Aires) in 2012 using experimental oral infections with DENV-2. Mosquitoes were incubated at 28°C and examined at 14 and 21 days p.i. to access viral dissemination and transmission. Batches of the Buenos Aires mosquitoes were also incubated at 15°C and 20°C.

**Results:**

Although mosquitoes from temperate Uruguay and Argentina were competent to transmit DENV, those from subtropical Argentina were more susceptible, displaying the highest virus titters in the head and presenting the highest dissemination of infection and transmission efficiency rates when incubated at 28°C. Interestingly, infectious viral particles could be detected in saliva of mosquitoes from Buenos Aires exposed to 15°C and 20°C.

**Conclusions:**

There is a potential risk of establishing DENV transmission in Uruguay and for the spread of dengue outbreaks to other parts of subtropical and temperate Argentina, notably during spring and summer periods.

## Background

Since the 1980’s, mostly due to the re-colonization of tropical and subtropical regions in the New World by *Ae. aegypti*, dengue has become the most formidable mosquito borne disease in South America, with more than 1 million clinical cases (280,000-718,000 laboratory confirmed/year) annually reported in the last years [1]. From 1998 to 2012, almost 80% of the dengue fever (DF) cases reported in South America were due to infections acquired in countries of the Southern Cone, the region that consists of Argentina, Brazil, Chile, Paraguay and Uruguay (Additional file 1: Figure S1, Additional file 2: Figure S2), Brazil being responsible for 97.5%, with 565,000 cases in 2012. Also, Paraguay has experienced important dengue outbreaks, with 39,000-42,000 cases informed in 2011–2012 and a report of 134,000 cases until May 2013. All four serotypes of dengue virus (DENV) circulate in Brazil and Paraguay, with recurrent severe epidemics. Not only neighboring Southern Cone countries, such as the two southernmost, Argentina and Uruguay, have suffered from the influence of the epidemiological situation in Brazil and Paraguay, but also Bolivia which has reported 26–42 thousand cases in the last three years.

Argentina reported around 4,700 DF cases between 1997 and 2007 (Additional file 2: Figure S2), mainly located in the northern provinces and involving DENV serotypes 1, 2 and 3 [2]. The first epidemic with national scope occurred in summer 2009, with 26,000 cases of DENV-1, reaching the country’s capital, Buenos Aires, in temperate Argentina [3,4]. During 2010, an outbreak of DENV-1 with more than 800 cases was documented in the northern province of Misiones, and isolated autochthonous cases due to DENV-1, -2 and −4 (unprecedented in the country) were reported in Chaco, Jujuy, Salta, Santa Fe, Santiago del Estero and Buenos Aires [5]. Since then, dengue transmission has been constant every summer from the center to the north of the country, but very few cases were confirmed by national health authorities: 81 cases of DENV-1, -2 in Santa Fe and Salta during 2011 [6] and 109 of DENV-2, -3 in La Rioja, Buenos Aires and Salta during 2012 [7]. Throughout the first quarter of 2013, a total of 155 autochthonous cases were notified, with a circulation of DENV-4 in Salta and Cordoba, DENV-1 in Cordoba and DENV-2 in Chaco and Buenos Aires [8]. In short, despite annual variations registered, the circulation of the four DENV serotypes from temperate to subtropical Argentina is currently a main public health concern. On the other hand, dengue transmission has not been described in Uruguay since the last autochthonous dengue cases were reported in 1916, in Salto [9]. However, imported dengue cases have annually been recorded in Uruguay since the late 1990’s, with 80–200 annually diagnosed in the last seven years [10]. These cases were due to DENV-1, acquired in Brazil and Paraguay, and DENV-1, -2 -3 imported from other South American countries [10].

After the 1960s eradication campaign achieved in the Americas, *Ae. aegypti* was reintroduced in Argentina in 1986 from the northwestern boundaries of the country, most probably from Bolivia and Brazil [11]. The geographic distribution has been expanded since the last update of the mosquito records by Vezzani & Carbajo [2]: towards the west, from Guaymallén, in Mendoza Province, to eight other counties of the province [12] and five localities in San Luis Province [13], previously free of *Ae. aegypti*. A similar expansion was observed to the south, in the provinces of La Pampa [14] and Buenos Aires [15]. Unexpectedly, *Ae. aegypti* has also been found in Neuquén city (38°57’S, 68°03’W), the southernmost registration of the mosquito in the globe [16].

*Ae. aegypti* had been absent from Uruguay for around 40 years when it was detected again in the country in 1997 [9]. Probably due to the geographical and climatic conditions in Uruguay, *Ae. aegypti* recolonization and dissemination was initially limited to those provinces closer to the warmer Argentinian and Brazilian borders [17]–[19]. *Ae. aegypti* is now present in ten out of 19 Uruguayan provinces, including the federal district of Montevideo, although those located in the northwestern boundaries with Argentina are the most infested, namely Salto and Paysandú, where the house index corresponding to the percentage of larvae or pupae infested dwellings may exceed 10% [19].

Although *Ae. albopictus* is well spread in neighboring Brazil, its distribution in Argentina and Uruguay is quite limited, in Argentina being restricted to a small part in the north of the province of Misiones [2]. In Uruguay, only few *Ae. albopictus* have been detected, once in the northern provinces of Rivera and Artigas near the Brazilian border [10]. Only *Ae. aegypti* has been incriminated as dengue vector in Argentina as well as in the Americas [2,20].

The assessment of *Ae. aegypti* population vector competence greatly contributes to the understanding of dengue transmission dynamics and consequently, the elaboration of mosquito control activities at local and regional levels. In South America, studies on vector competence for DENV have been only limited to Brazilian and French Guyanese populations of *Ae. aegypti*[21,22]. Even though data on dengue incidence in Argentina during the last two decades has been available, with a large heterogeneous annual incidence between northern sub-tropical and southern temperate zones, the vector competence for DENV of *Ae. aegypti* populations remains unknown, and no information on the vectorial status for DENV of *Ae. aegypti* from Uruguay is available. Herein, we assess the vector competence for DENV-2 of *Ae. aegypti* populations from Argentina and Uruguay, including samples from the southernmost area with dengue transmission in the continent.

## Methods

**Figure 1:**
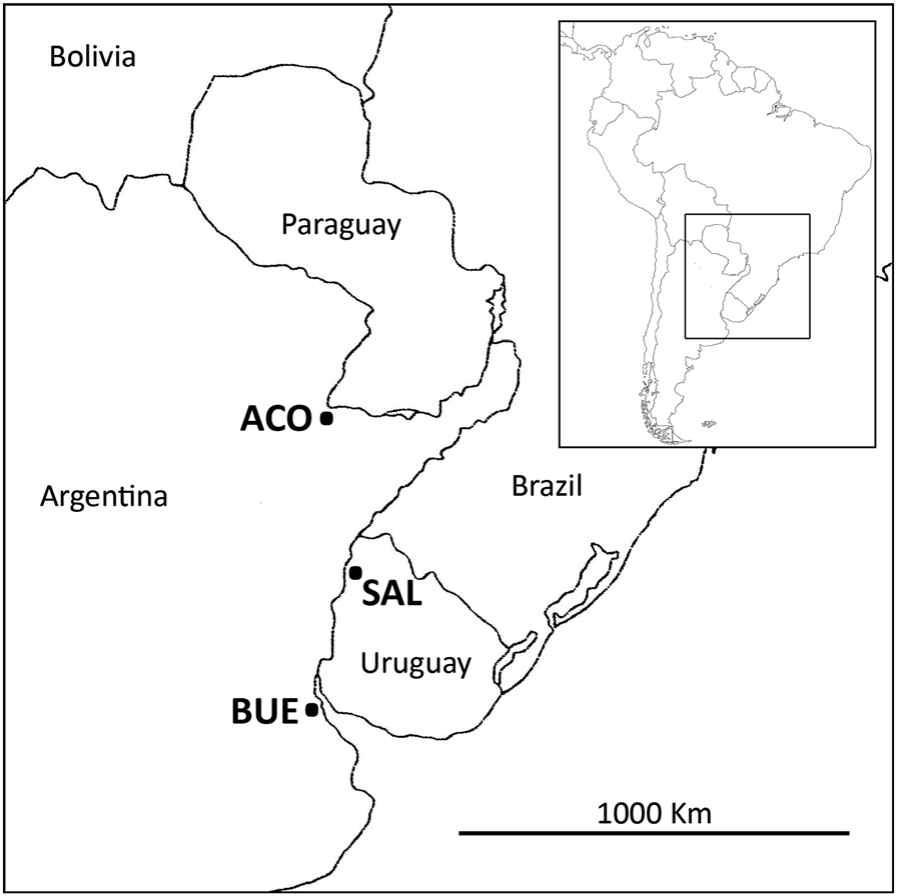
**Location of *Aedes aegypti *** **populations sampled.**

The mosquitoes used in this study were *Ae. aegypti* from two localities in Argentina, Buenos Aires (BUE) (34°35’ 58°22 W) and Corrientes (ACO) (27°28’S 58°50’W), and one locality in Uruguay, Salto (SAL) (31°23’S 57°58’W) (Figure 1). The mosquitoes were collected from March to May 2012 with ovitraps (N = 10–58) set in different districts of each locality: Ranelagh, Vicente Lopez, Lomas de Zamora and Quilmes for BUE, Camba Cua and Centro for ACO and all districts for SAL. The eggs gathered in all districts of the same locality were pooled.

Afterwards, field collected eggs were hatched and reared in insectaries (24 ± 1°C; 70 ± 10% RH; 14 h:10 h light:dark cycle). Larvae were maintained in pans (~100 larvae/pan) containing 1 liter of dechlorinated tap water supplemented with yeast tablets. Obtained adults were identified [20], exclusively consisting of *Ae. aegypti*. F0 generation *Ae. aegypti* adult males and females were kept together for approximately three weeks. They were provided daily with 10% sucrose solution and fed on mice every two days for obtaining the F1 generation eggs. The F1 generation was reared under the same conditions. All experimental oral infections were performed with mosquitoes from the F1 generation. The day before the infectious blood-meal, batches of 65 females of ~5 days of emergence were isolated in feeding boxes and starved for 24 hs. Mosquitoes were then exposed to the infectious blood-meal containing a final viral titer of 10^7^ focus forming units (ffu)/mL which consists of a mixture of two parts of washed rabbit erythrocytes and one part of the viral suspension added with a phagostimulant (0.5 mM ATP). Viral suspension consisted of the supernatant obtained from a 5–8 day incubation on a C6/36 *Ae. albopictus* culture cell infected with a reference strain of DENV-2 isolated from a human case in Thailand in 1974 [23]. Mosquito feeding was limited to 50 min. After the infectious blood-meal, non-engorged females were discarded. Fully engorged females were incubated at 28°C constant temperature, 70 ± 10% RH and 14 h:10 h light:dark cycle, with daily access to 10% sucrose solution. Essentially, samples of 30–46 mosquitoes of each population were examined at days 14 and 21 post-infection (pi). The effect of temperature on vector competence was also tested with batches of BUE mosquitoes exposed to the same infectious blood meal and kept as described above but incubated at constant temperatures of 15°C and 20°C.

For the determination of viral dissemination, mosquito heads were individually ground in 250 μL Leibovitz L15 medium (Invitrogen) supplemented with 4% Fetal Bovine Serum (FBS) and then centrifuged at 10,000 rpm for 5 min at +4°C. Viral titer in heads was estimated by focus fluorescent assays. Serial dilutions of the obtained supernatant were inoculated onto a C6/36 *Ae. albopictus* cell monolayer in 96-well plates, and incubated at 28°C for 5 days. Plates were fixed with 3.6% formaldehyde, washed three times with PBS and analyzed by indirect immunofluorescence assay (IFA) with hyperimmune ascetic fluid specific to DENV-2 as the primary antibody and fluorescein-conjugated goat anti-mouse as the second antibody (Biorad). The disseminated infection rate (DIR) corresponded to the proportion of mosquitoes with virus detected in heads among tested ones.

In order to assess the transmission rate (TR) and transmission efficiency (TE), mosquito saliva was collected in individual pipette tips containing 5 μL FBS. After 30 min of salivation, FBS containing saliva was added to 45 μL Leibovitz L15 medium. Mosquito saliva was inoculated onto a C6/36 cell monolayer in 96-well plates and examined after incubation as described above. TR corresponds to the proportion of mosquitoes with infectious saliva among mosquitoes able to ensure viral dissemination beyond the midgut barrier, that is, among those whose head homogenates were positive in focus fluorescent assays on C6/36 *Ae. albopictus* cells. TE represents the proportion of mosquitoes with infectious saliva among those exposed to the infectious blood meal and examined by focus fluorescent assays on C6/36 *Ae. albopictus* cells.

To compare the number of infectious viral particles, the Wilcoxon signed rank test was adopted to analyze pairwise comparison at days 14 and 21 pi for each mosquito population and the Kruskal-Wallis test for comparison among several populations. To compare proportions (DIR, TR and TE), the Chi square test was used to analyze pairwise comparison at days 14 and 21 pi for a given mosquito population or among several populations. Significant difference was established when p-values were lower than 0.05. Data analyses were conducted with PRISM 5.0 software (GraphPad Software, San Diego-CA, USA, 2007).

## Results

All tested *Ae. aegypti* populations from both subtropical and temperate Argentina and Uruguay were susceptible and competent to transmit DENV-2. In general, the ACO population from northern subtropical Argentina performed the best measures of DIR, TR and TE compared to the temperate populations from both Argentina (BUE) and Uruguay (SAL) when incubated at 28°C (Table 1). The DIR determined by fluorescent assay of head homogenates on C6/36 cells varied from 53.3% (ACO, SAL) to 66.7% (BUE) at day 14 pi, and from 76.7% (ACO, SAL) to 78.8% (BUE) at day 21 pi. When comparing DIR estimated at 14 and 21 days pi for each individual mosquito population, no significant difference was detected (Chi square test: p > 0.05). Moreover, when comparing DIR at day 14 pi and day 21 pi for all three populations, no significant difference was apparent (Chi square test: p > 0.05). Estimations of DIR by head squashes stained by IFA were also performed and similar results were essentially obtained (Additional file 3: Table S1).

**Table 1:** **Disseminated infection rate (DIR), transmission rate (TR) and transmission efficiency (TE) to DENV-2 of *Ae. aegypti *** **from Argentina and Uruguay after 14 and 21 days at 28°C**

**Days post-infection**	**Argentina**						**Uruguay**		
	**BUE**			**ACO**			**SAL**		
	**DIR**	**TR**	**TE**	**DIR**	**TR**	**TE**	**DIR**	**TR**	**TE**
**14**	66.7	20	10.5	53.3	31.3	18.5	53.3	37.5	20
	(30)	(20)	(38)*	(30)	(16)	(27)*	(30)	(16)	(30)
**21**	78.1	8	6.7	76.7	34.8	36.4	76.7	21.7	17.9
	(32)	(25)	(30)*	(30)	(23)	(22)*	(30)	(23)	(29)*

BUE: Buenos Aires; ACO: Corrientes; SAL: Salto; DIR: Disseminated infection rate is the proportion of mosquitoes with virus detected in the head among examined ones; TR: Transmission rate is the proportion of mosquitoes with infectious saliva among mosquitoes able to disseminate the virus beyond the midgut barrier; TE: Transmission efficiency is the proportion of mosquitoes with infectious saliva among the examined ones; In parenthesis for DIR and TE is the number of mosquitoes examined; In parenthesis for TR is the number of mosquitoes able to disseminate the infection beyond the midgut. * Due to technical issues, particularly fungal contamination of inoculated cell cultures, the number of examined saliva to determine TE was sometimes distinct from the total number of examined mosquitoes for DIR.

Virus was detected by focus fluorescent assay on C6/36 *Ae. albopictus* cells.

**Figure 2:**
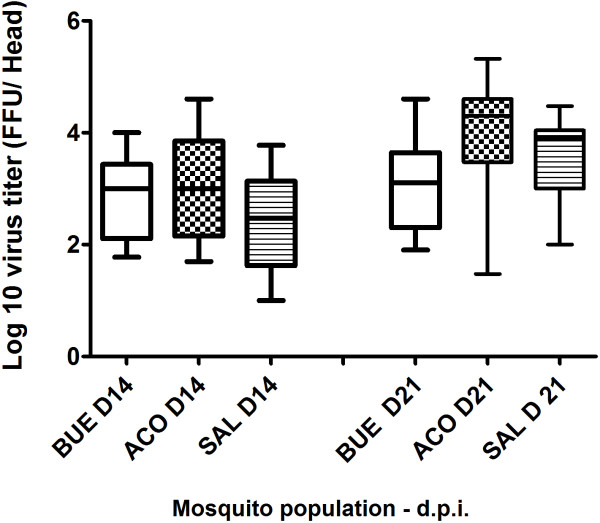
**DENV-2 load in heads of *Ae. aegypti *from Buenos Aires (BUE) and Corrientes (ACO) in Argentina and Salto (SAL) in Uruguay, after 14 and 21 days at 28°C.** Virus was detected by focus fluorescent assay on C6/36 *Ae. albopictus* cells.

When comparing the 3 populations examined at a given day pi, the virus titer of head homogenates was similar (Kruskal-Wallis test: p > 0.05). The median virus titer in mosquito heads tended to be higher at day 21 pi than at day 14 pi (Figure 2). However, when considering each mosquito population, viral titer was only significantly higher at day 21 pi than at day 14 pi for ACO (t test: p < 0.05). The highest viral loads detected in a mosquito head were exhibited by individuals from the ACO population regardless of the time of incubation.

To determine TR and TE, the virus was detected in mosquito saliva following inoculation onto C6/36 cell cultures. TR varied from 20% (BUE) to 37.5% (SAL) at day 14 pi, and from 8% (BUE) to 34.8% (ACO) at day 21 pi (Table 1). In addition, TE ranged from 10.5% (BUE) to 20% (SAL) at day 14 pi, and from 6.7% (BUE) to 36.4% (ACO) at day 21 pi (Table 1). As a rule, TR was higher than TE. TR and TE were mostly similar among all three mosquito populations at day 14 (Chi square test: p > 0.05). At day 21 pi, the temperate population of BUE depicted the lowest TE (Chi square test: p < 0.05).

In short, even though not statistically significant (Chi square test: p > 0.05), DIR, TR and TE at day 21 pi were essentially higher than at day 14 pi when incubated at 28°C. Similar results were also observed when BUE mosquitoes were incubated at lower temperatures (15°C and 20°C) (Table 2). This result suggests that dissemination and transmission did not significantly change between 14 and 21 days pi at a given incubation temperature.

**Table 2:** **Effects of temperature on disseminated infection rate (DIR), transmission rate (TR) and transmission efficiency (TE) to DENV-2 of *Ae. aegypti *** **from Buenos Aires (Argentina) after 14 or 21 days at 15°C, 20°C or 28°C**

**Days post-infection**	**Buenos Aires**								
	**15°C**			**20°C**			**28°C**		
	**DIR**	**TR**	**TE**	**DIR**	**TR**	**TE**	**DIR**	**TR**	**TE**
**14**	29.4	0	0	16.7	33.3	10	66.7	20	10.5
	(17)	(5)	(17)	(24)	(4)	(20)	(30)	(20)	(38)*
**21**	7.7	100	7.7	22.7	100	29.4	78.1	8	6.7
	(13)	(1)	(13)	(22)	(5)	(17)	(32)	(25)	(30)*

DIR: Disseminated infection rate is the proportion of mosquitoes with virus detected in the head among examined ones; TR: Transmission rate is the proportion of mosquitoes with infectious saliva among mosquitoes able to disseminate the virus beyond the midgut barrier; TE: Transmission efficiency is the proportion of mosquitoes with infectious saliva among the examined ones; In parenthesis for DIR and TE is the number of mosquitoes examined; In parenthesis for TR is the number of mosquitoes able to disseminate the infection beyond the midgut. * Due to technical issues, particularly fungal contamination of inoculated cell cultures, the number of examined saliva to determine TE was sometimes distinct than the total number of examined mosquitoes for DIR.

Virus was detected by fluorescent assay on C6/36 *Ae. albopictus* cells.

**Figure 3:**
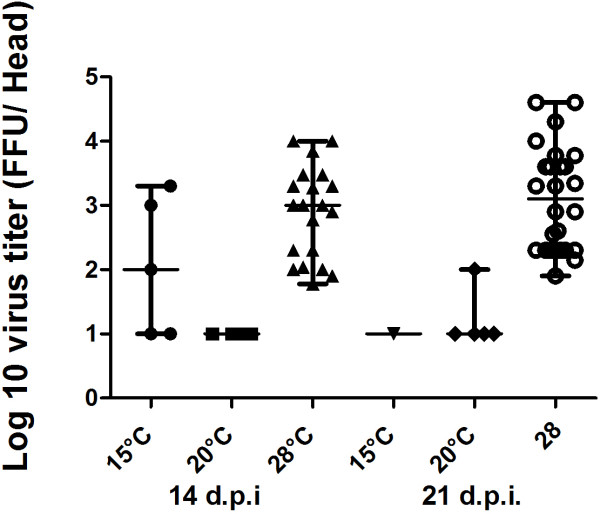
**Effects of temperature on DENV-2 load in heads of *Ae. aegypti *from Buenos Aires (Argentina), after 14 and 21 days at 15°C, 20°C or 28°C.** Virus was detected by focus fluorescent assay on C6/36 *Ae. albopictus* cells.

Both DIR and viral titer in mosquito heads were markedly higher when BUE mosquitoes were incubated at 28°C than at 15°C and 20°C (Chi square test: p < 0.05) (Table 2, Figure 3). Intriguingly, viral dissemination was recorded both at 14 days pi in BUE mosquitoes exposed to 15°C and 20°C, although DIRs were two to ten times lower than when the same mosquito population was incubated at 28°C (Table 2). However, TE was higher in BUE mosquitoes incubated at 20°C, especially when examined at day 21 pi (Chi square test: p < 0.05). The amount of positive saliva was scanty, regardless of mosquito population, incubation time or temperature (Tables 1 and 2), hampering statistical analysis. Remarkably, infectious viral particles were detected 21 days after the infectious blood-meal in saliva of one BUE *Ae. aegypti* incubated at 15°C (Table 2). Viral loads in positive saliva were essentially low and similar (median = 0.8 ffu/saliva; range = 0.8 – 2.4 ffu/saliva) among mosquito populations regardless of incubation time and temperature, except for a single BUE mosquito at 14 days pi incubated at 28°C (12 ffu/saliva).

## Discussion

We have herein demonstrated for the first time that *Ae. aegypti* populations from Uruguay and Argentina are competent in experimental DENV-2 transmission, and that female mosquitoes sampled in the subtropics (ACO) are more efficient than those of temperate regions (SAL and BUE), displaying the highest virus titers in the head and presenting the highest dissemination of infection and transmission efficiency rates. Interestingly, the population from Salto, a dengue-free temperate area of Uruguay, was more efficacious to transmit DENV than mosquitoes from Buenos Aires (temperate Argentina) where dengue transmission has been confirmed since 2007. Noteworthy is the lower transmission rate of *Ae. aegypti* from temperate Argentina (BUE) despite a high viral dissemination, which may suggest an efficient barrier to virus delivery from salivary glands. Extending the incubation period from 14 to 21 days pi did not increase transmission even if viral dissemination tended to slightly augment. There was an exception with respect to the ACO population from subtropical Argentina with an enhanced transmission from day 14 to day 21 pi and the highest virus titers in heads. Curiously, infectious viral particles could be detected in saliva of mosquitoes from temperate Argentina exposed to lower constant temperatures [15°C (21 dpi) and 20°C (14 and 21 dpi)]. This is a remarkable finding as dissemination and transmission rates of DENV are unusually very low to null in *Ae. aegypti* incubated at constant temperatures ≤ 20°C [24]–[26]. Nevertheless, it has been recently demonstrated that a great diurnal temperature fluctuation regime at an average of 20°C increases DIR and reduces the extrinsic incubation period of DENV in *Ae. aegypti* compared to a constant exposition at 20°C. Unexpectedly therefore, a low average temperature with great fluctuations could favor transmission in temperate *Ae. aegypti* populations [26].

In South America, Brazil, Bolivia and Paraguay have been used to dengue outbreaks since the late 1980s, whereas dengue transmission is usually low in most regions of Argentina and totally absent in Uruguay. Thus, it has been hypothesized that vectorial capacity for DENV of *Ae. aegypti* populations from these two southernmost countries would be low enough to limit the transmission [19,20]. This parameter is influenced mostly by mosquito density and vector competence as well as the extrinsic incubation period, which in turn, is strongly regulated by temperature [2,19,24,27]. Therefore, in this paper, we assessed one of the factors determining vectorial capacity (i.e. vector competence) in different *Ae. aegypti* populations of Argentina and Uruguay*.*

Vector competence is a quantitative phenotype that defines the degree of compatibility between a vector population and a pathogen such as DENV. It is controlled by multiple mosquito genes [28,29] and is dependent upon environmental factors [20,22,29]. Interestingly, *Ae. aegypti* populations from Buenos Aires and Corrientes in Argentina as well as Salto in Uruguay are not very genetically differentiated, suggesting an important gene flow between populations from the Southern Cone [11,18,30]. Collectively, data on genetic population studies implied that *Ae. aegypti* of Eastern Argentina and Uruguay, including our three study areas, are essentially genetically homogeneous and pointed out that Southern Brazilian and Paraguayan populations are import sources for colonization and genetic exchange with Argentine gene pools, which in turn has an important role in colonizing Western Uruguay, where Salto is located [11]. Commercial traffic can enhance human displacement, facilitating mosquito migration, increasing the risk of dengue spreading to Uruguay and Argentina [11,18].

In addition to the vector competence, temperature plays a determinant role in vectorial capacity. Indeed, temperature influences viral replication in mosquitoes, mosquito immature stage development, oviposition and therefore, mosquito density. As Uruguay and Central Eastern Argentina possess a temperate climate, we investigated whether dissemination and transmission of DENV are likely to occur at low temperatures. In doing so, we incubated orally DENV-infected *Ae. aegypti* from Buenos Aires (BUE) at two low constant temperatures, 15°C and 20°C. These temperatures correspond to average indexes recorded during the fall and spring, in Buenos Aires. We found that although dissemination of DENV-2 was markedly lower in mosquitoes incubated at 15°C and 20°C in comparison to those maintained at 28°C, the transmission efficiency was higher in the mosquitoes incubated at 20°C than at 15°C and 28°C, especially at 21 days pi. Although virus titers in *Ae. aegypti* infected female heads were significantly higher in mosquitoes incubated at 28°C, they could also reach substantial levels in some mosquitoes exposed at 15°C and 20°C. Most importantly, infectious viral particles were detected in saliva of mosquitoes incubated at 15°C (at 21 days pi) and 20°C (at 14 and 21 days pi). Nevertheless, the number of viral particles necessary to induce vertebrate host infection remains unknown. It has been proposed that the available methods to detect and determine titers of infectious viral particles in the saliva of experimentally dengue infected *Ae. aegypti* certainly provide neither an exact picture of saliva infectivity nor real transmission efficiency in nature [31]. Besides, viral dissemination is a reliable proxy as it is positively correlated with the presence of viral particles in saliva [32,33]. Accordingly, transmission values [TR and TE] of our tested populations are probably much higher than expected. Therefore, *Ae. aegypti* from temperate South Cone regions, like in Buenos Aires and Salto, are expected to be capable to transmit DENV even when average temperatures drop to 20°C and 15°C. Actually, vector competence of the tested Argentine and Uruguayan mosquito samples was similar to those of several *Ae. aegypti* populations sampled in dengue epidemic Brazilian localities tested for the same DENV-2 strain previously [22]. Probably, the most limiting factors related to DENV transmission are the mosquito survival and biting rates especially during the lower temperature periods, which are unknown in natural conditions for temperate South American populations [2].

The area embracing Buenos Aires and its peripheral districts (i.e. BUE in our sampling) is the southernmost point with dengue transmission in the Americas. Although dengue epidemics have been recorded in subtropical northern Argentina since 1997, the first cases of dengue (DENV-3) were documented in Buenos Aires in February 2007, coinciding with the detection of 158 imported cases, mainly people returning from Paraguay [34]. Afterwards, 105 autochthonous cases of DENV-1 (year 2009), 13 of DENV-2 (2010 and 2013) and 19 of DENV-3 (2012) were registered [5,7,8,35]. Long after the large scale eradication program of *Ae. aegypti*, the species reinvaded the city of Buenos Aires in 1995 [36]. It quickly became one of the most abundant mosquito species encountered in artificial containers, as eggs and larvae mostly in flower vases in cemeteries as well as discarded tires, and as adults in private premises in Buenos Aires and several urban settings in Argentina [37]–[40]. In Buenos Aires, *Ae. aegypti* female activity is mainly restricted from October to May (i.e. the spring-fall period), peaking in March, when most autochthonous cases of dengue have been recorded [2,41,42].

Currently, Uruguay is the only country in South America without any local dengue transmission [1]. We have herein demonstrated that the tested Uruguayan *Ae. aegypti* population is experimentally competent to DENV transmission. Evidently, the dengue transmission risk assessment in Uruguay depends not only upon the evaluation of multiple components determining vectorial capacity, of which vector competence is merely one, but also upon ecological and climatic factors. The climate in Uruguay is temperate, with average annual temperatures between 16°C in Montevideo in the south to around 19°C in Salto in the west where temperatures can drop to −2°C/-5°C during the fall-winter period [43]. Then, larval development and adult activities such as oviposition and blood-seeking can slow down [19,20]. On the other hand, the absolute maximum temperature may reach 42°C during summer time (Dec-Mar) or 20°C-31°C even in the fall-winter. Thus, these climatic conditions may restrain, but not prevent, vector development and activity during some part of the year. Actually, the house index remains high enough in Salto to sustain dengue transmission [19]. The occurrence of dengue transmission in Uruguay may be a matter of time, as it was the case of Buenos Aires [2,18,34,35].

## Conclusion

To conclude, frequent movements of both mosquitoes and dengue cases between South American endemic/epidemic regions and Uruguay and Argentina combined with substantial vector competence for DENV of *Ae. aegypti* from subtropical and temperate areas demonstrated herein, exemplifies the actual risk of establishing DENV transmission in Uruguay and the spread of dengue outbreaks to other parts of subtropical and temperate Argentina as well as other dengue-free areas in the Southern Cone countries.

## Competing interests

The authors declare that they have no competing interests.

## Authors’ contribution

RLO conceived the study, carried out experimental infections of mosquitos, performed titration assays and drafted the manuscript. AVR participated in tritration assays and to revise the manuscript. DV and GV conducted mosquito collections and helped to draft the manuscript. LM and MV participated in tritration assays. ABF helped to draft and to revise the manuscript. All authors read and approved the final version of the manuscript.

## Pre-publication history

The pre-publication history for this paper can be accessed here:

http://www.biomedcentral.com/1471-2334/13/610/prepub

## Supplementary Material

Additional file 1: Figure S1 Dengue cases clinically diagnosed in South America in 1998–2013, according to countries belonging to the Southern Cone. Source: OPAS (2013). Click here for file

Additional file 2: Figure S2 Annual incidence of dengue cases in South American countries of Southern Conne (1995–2012). Click here for file

Additional file 3: Table S1 Disseminated infection rate (DIR) of DENV-2 in *Aedes aegypti* from Buenos Aires (BUE) and Corrientes (ACO), Argentina, and Salto (SAL), Uruguay. DIR was comparatively determined by the examination of head squashes (HS) and inoculation of head homogenates onto C6/36 *Ae. albopictus* cells (CC). For head squashes, mosquito heads were detached and squashed between glass slides, fixed in acetone (20 min/4°C) and individually analyzed by indirect immunofluorescence assay (IFA) using hyperimmune ascetic fluid specific to DENV-2 as primary antibody and fluorescein-conjugated goat anti-mouse as second antibody. Saliva was not collected from mosquitoes whose DIR was determined by examination of HS. To detect focus-forming units in head homogenates, heads were individually ground and serial dilutions of the obtained supernatant were inoculated onto C6/36 cells monolayer in 96-well plates, and incubated at 28°C for 5 days. Plates were analyzed by IFA. Click here for file
