# Supplementary figures and images for: Aedes aegypti from temperate regions of South America are highly competent to transmit dengue virus
Source: BMC Infect Dis. 2013 Dec 28;13:610. doi: 10.1186/1471-2334-13-610 (PMC3929315; doi:10.1186/1471-2334-13-610)

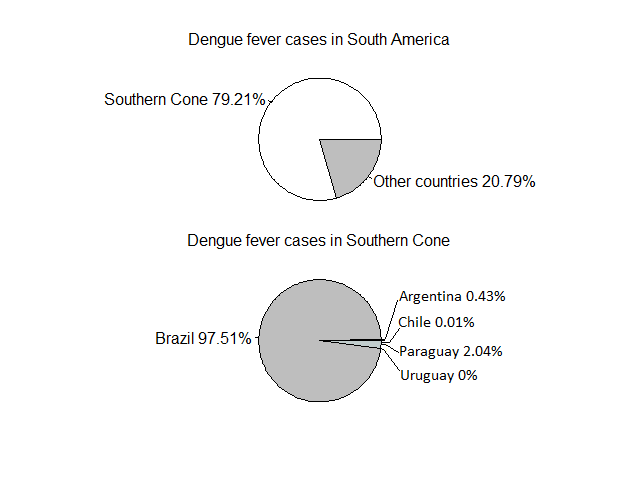

Supplement: Additional file 1: Figure S1 — Dengue cases clinically diagnosed in South America in 1998–2013, according to countries belonging to the Southern Cone. Source: OPAS (2013). [file 1471-2334-13-610-S1.tiff]

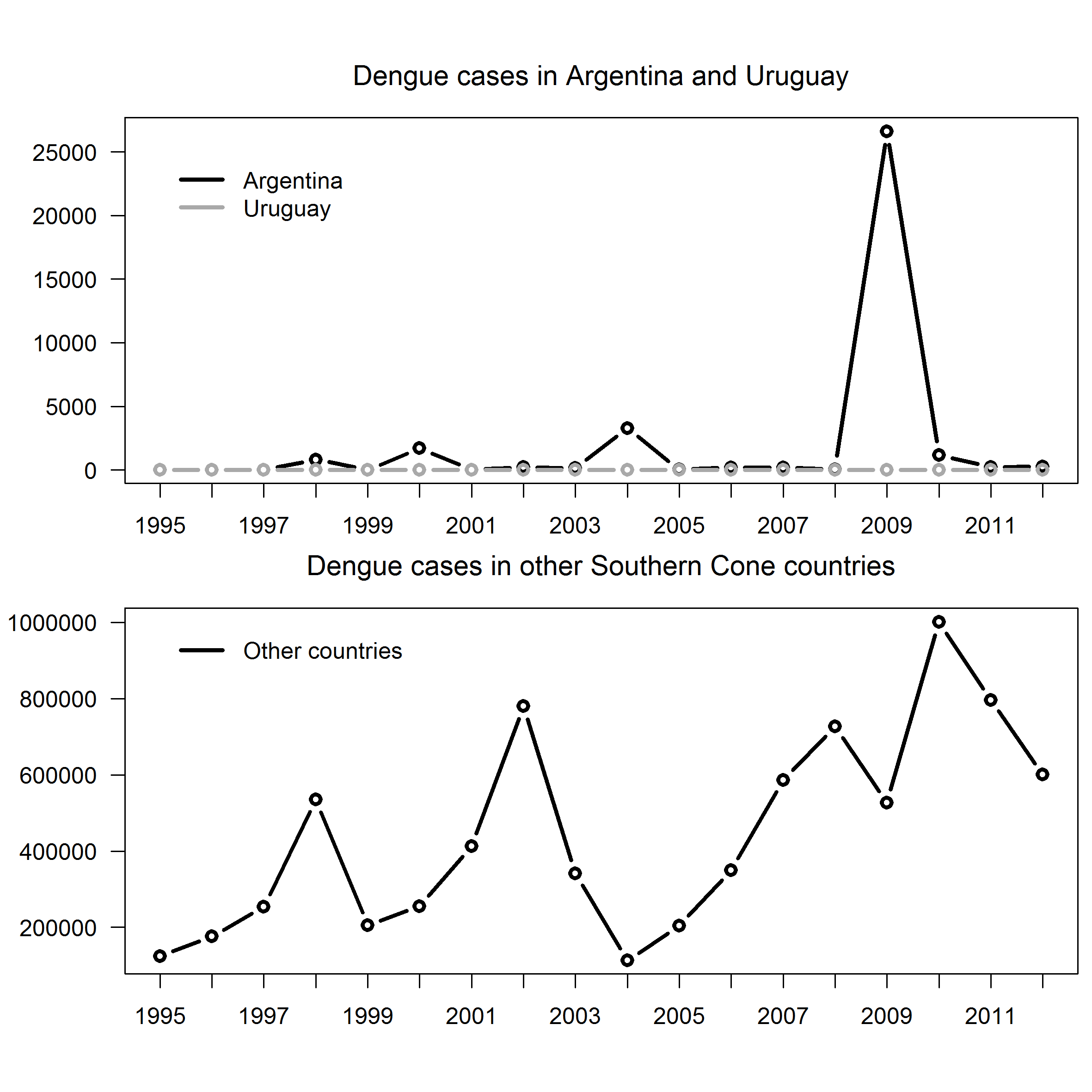

Supplement: Additional file 2: Figure S2 — Annual incidence of dengue cases in South American countries of Southern Conne (1995–2012). [file 1471-2334-13-610-S2.tiff]
