# Supplementary material for: Aedes aegypti from temperate regions of South America are highly competent to transmit dengue virus
Source: BMC Infect Dis. 2013 Dec 28;13:610. doi: 10.1186/1471-2334-13-610 (PMC3929315; doi:10.1186/1471-2334-13-610)
Supplement: Additional file 3: Table S1 — Disseminated infection rate (DIR) of DENV-2 in Aedes aegypti from Buenos Aires (BUE) and Corrientes (ACO), Argentina, and Salto (SAL), Uruguay. DIR was comparatively determined by the examination of head squashes (HS) and inoculation of head homogenates onto C6/36 Ae. albopictus cells (CC). For head squashes, mosquito heads were detached and squashed between glass slides, fixed in acetone (20 min/4°C) and individually analyzed by indirect immunofluorescence assay (IFA) using hyperimmune ascetic fluid specific to DENV-2 as primary antibody and fluorescein-conjugated goat anti-mouse as second antibody. Saliva was not collected from mosquitoes whose DIR was determined by examination of HS. To detect focus-forming units in head homogenates, heads were individually ground and serial dilutions of the obtained supernatant were inoculated onto C6/36 cells monolayer in 96-well plates, and incubated at 28°C for 5 days. Plates were analyzed by IFA. [file 1471-2334-13-610-S3.doc]

**Table S1.** Disseminated infection rate (DIR) of DENV-2 in *Aedes aegypti* from Buenos Aires (BUE) and Corrientes (ACO), Argentina, and Salto (SAL), Uruguay. DIR was comparatively determined by the examination of head squashes (HS) and inoculation of head homogenates onto C6/36 *Ae. albopictus* cells (CC). For head squashes, mosquito heads were detached and squashed between glass slides, fixed in acetone (20 min/4°C) and individually analyzed by indirect immunofluorescence assay (IFA) using hyperimmune ascetic fluid specific to DENV-2 as primary antibody and fluorescein-conjugated goat anti-mouse as second antibody. Saliva was not collected from mosquitoes whose DIR was determined by examination of HS. To detect focus-forming units in head homogenates, heads were individually ground and serial dilutions of the obtained supernatant were inoculated onto C6/36 cells monolayer in 96-well plates, and incubated at 28°C for 5 days. Plates were analyzed by IFA.

| Days  p.i. | ARGENTINA | | | | URUGUAY | |
| --- | --- | --- | --- | --- | --- | --- |
|  | BUE | | ACO | | SAL | |
|  | HS | CC | HS | CC | HS | CC |
| 14 | 64.4  (45) | 66.7  (30) | 80  (30) | 53.3  (30) | 51.6  (31) | 53.33 (30) |
| 21 | 73.3  (30) | 78.8  (32) | 88.9  45) | 76.7  (30) | 69.6  (46) | 76.7  (30) |

Disseminated infection rate is the proportion of female mosquitoes with virus detected in the head among the examined ones (in parenthesis).
